# Supplementary material for: What If…? Pandemic policy-decision-support to guide a cost-benefit-optimised, country-specific response
Source: PLOS Glob Public Health. 2022 Aug 24;2(8):e0000721. doi: 10.1371/journal.pgph.0000721 (PMC10022143; doi:10.1371/journal.pgph.0000721)
Supplement: Attachment — Submitted filename: PLOS_WhatIf_responsereviewers.pdf [file pgph.0000721.s011.pdf]

RE: Response to Reviewers. PLoS Global Public Health. PGPH-D-21-01132

What If...? Pandemic Policy-Decision-Support to Guide a Cost-Benefit-Optimised, Country-Specific Response

Dear editor,

We kindly thank you for the effort involved to find reviewers for this work.

Most questions raised are related to a single misunderstanding, which has now been clarified in the text.

In short, the reviewers assumed that our work was either predicting or dependent upon the association of the reported metric used in our models (for instance R_E) with the "*real"* value.

This work specifically does not comment on the association between the reported metric and the actual ground truth of that metric. To answer that question would require large-scale coordinated primary data collection. Rather, we estimate how these reported metrics (irrespective of their association with reality) would change in light of reported policy changes.

As many policymakers use these metrics to measure *relative* policy effectiveness (usually with an understanding of their relationship to reality). Our work aligns with the information that policymakers use routinely.

We greatly appreciate the opportunity to clarify this important point which was not well communicated in our original manuscript.

Additionally, reviewing the text, we found that the *Author Summary* over-simplified the findings, which may have been the source of confusion. This section is now rewritten.

The revised manuscript is attached with edits in blue.

Please find our point-by-point responses below.

Dr Mary-Anne Hartley (MD, PhD, MPH)
Intelligent Global Health Research Group
EPFL (Swiss Institute of Technology)
mary-anne.hartley@epfl.ch
+41788667160

Reviewer 1

Comment: The described application looks like one of the potentially more useful results of the pandemic, especially in that it has potential to be useful in future epidemics.

Reply: We thank the reviewer for seeing the large scale potential of this work for public health policy decision support. We share the view that it could provide an analytical scaffold for future epidemics and look forward to seeing others build on this work.

Comment: My only comment is with regards to the data sources- as with any multi-country COVID-19 estimation project, the crux lies with being able to adjust for the systemic bias created by differences in the completeness of data reporting, in particular between LMIC and HIC.

- **R(E)** It is not clear to me how the authors corrected for this, in particular with regards to the case data informing R(e). (The efforts the authors took to exclude countries or periods with very low case data does not correct for this, as the problem does not automatically lead to very low cases reported, just to lower-than-should-be cases.)
- **OxCGRT:** Similarly the accuracy of the OxCGRT data hinges on these restrictions having been publicised and their publication found by the OU researchers, which additionally introduces bias.
- **Solution:** One solution might be to declare this limitation and caution that the application will be differentially useful for HIC and LMIC, something we have in fact grown used to (writing from an LMIC).

Reply: We thank the reviewer for this useful suggestion and agree that we should more prominently highlight this limitation.

The suggested change now appears prominently throughout the revised manuscript.

Now emphasised in abstract

> year of data (04/2020—06/2021) from 116 countries. The economic damage of the policies is also evaluated on the 29 higher income countries for which data is available.

With regards to the systematic biases in data collection between countries. Several of our authors are from LMICs in Africa and strongly share your interest on the impact of data representation in low-resource settings.

The data used to train the R_E model represents roughly 91% of the global population. The included countries have an average age of 33 years (compared to 30 years for the global population) and an average urban population of 60% (compared to 64% of the global population). The economic model is naturally limited to those countries with relatively regular and standardised reporting.

Added at line 108

> The retained countries represent roughly 90% of the global population across a wide variety of sociodemographic settings from each continent. It covers a GDP range of 1.4 to 17900 billion USD (measured on 31/12/2020) with an average urban population of 60% (compared with 64% globally according to the World Bank data sources).

We hope this work can advocate for the potential value of regular economic reporting in low-resource settings.

Detail added at line 116

Geographic scope of the UER model

Of the above selected countries, only 29/116 (25%) provided suitable labels that could be validated for the UER model. Indeed, unemployment rate is more challenging to collect and data quality standards (absence of null values, values up to 31/03/2021 collected and not forecasted), were only met in few countries. To ensure that we limited assumptions to the most represented subset, we focused on high income settings within close geographic proximity as a proof-of-concept, including 27 states from the European Union as well as neighboring Switzerland, Norway and the UK. When applying the same filters as above, only Poland was excluded due to missing key features. Thus, the economic component of this work is limited to higher income countries. We hope the results can serve advocate for the potential value of regular economic reporting in lower resource settings. This work is open source so that new data can be added as it becomes available.

Reviewer 2

Comment 1: Thank you to the authors for the manuscript and work involved in developing and analysing the modelling framework.

I have several major concerns with the model developed in the manuscript.

1. To develop an optimised cost-benefit response model for individual countries based on available data, is by definition not optimised. For many countries in the world, the data do not simply exist, or if they do are not necessarily representative, robust proxies of the situation they are seeking to measure. The authors realised this when developing the economic model where data were available for only 29 countries, and - that too HICs.

Reply: We thank the reviewer for these pertinent remarks, which highlight the importance to clarify this point in our manuscript.

As shown in the title, this work provides an analytical scaffold for *guiding* cost-benefit optimised country-specific policy response. To do so, it seeks to reveal how the data routinely used by policymakers as proxies of cost and benefit reacts to changes in policies.

Indeed "available data" is not universally available for all metrics across all countries with a perfect association to reality. We estimate that this will never be possible.
We use massive global-scale, open-access data sources, which are routinely used by policymakers (regardless of their association with reality).

We do not seek to estimate the *true* values of the metrics used but rather to estimate their relative values in light of reported policy changes.
We have now made this clear in the manuscript and thank the reviewer for the opportunity to do so.

Explanation added at line 363

There are several limitations that must be mentioned. Firstly, it is important to note that this work specifically does not comment on the association between the metric predicted and the actual ground truth of that metric. That question would likely require large-scale coordinated primary data collection. Rather, we estimate how this reported metric (irrespective of its association with reality) would change in light of reported policy changes. As many policymakers use these metrics to measure relative policy effectiveness (usually with an understanding of their relationship to reality), our work aligns thus attempts to align with the information that policymakers use routinely. Additionally, while our R_E model covers approximately 90% of the global population, we restrict the UER model to countries where economic reporting is available and likely to be interoperable. More data is needed to extend the geographic scope of this model and we hope that the encouraging results from this work may help advocate for more regular and reliable reporting in lower resource settings.

The data used to train the R_E model represents roughly 91% of the global population. The included countries have an average age of 33 years (compared to 30 years for the global population) and an average urban population of 60% (compared to 64% of the global population).

Added at line 108

The retained countries represent roughly 90% of the global population across a wide variety of sociodemographic settings from each continent. It covers a GDP range of 1.4 to 17900 billion USD (measured on 31/12/2020) with an average urban population of 60% (compared with 64% globally according to the World Bank data sources).

This model computes the *benefit* component of optimization and has global relevance. For cost, we show the potential of a subset of robustly reported data from a well known economic region.

Our open-source models can be complemented with new data as it becomes available.

Line 116

Geographic scope of the UER model

Of the above selected countries, only 29/116 (25%) provided suitable labels that could be validated for the UER model. Indeed, unemployment rate is more challenging to collect and data quality standards (absence of null values, values up to 31/03/2021 collected and not forecasted), were only met in few countries. To ensure that we limited assumptions to the most represented subset, we focused on high income settings within close geographic proximity as a proof-of-concept, including 27 states from the European Union as well as neighboring Switzerland, Norway and the UK. When applying the same filters as above, only Poland was excluded due to missing key features. Thus, the economic component of this work is limited to higher income countries. We hope the results can serve advocate for the potential value of regular economic reporting in lower resource settings. This work is open source so that new data can be added as it becomes available.

Comment 2:

2. Decision-making in health is not only a function of health variables, environmental variables and 1 or 2 economic variables. Multi-tiered processes are in place with several stakeholders, international pressure often plays a large role, but perhaps the most important feature is that a country could have best ‘optimised’ policies, but implementation is out of the hands on the policymakers. Therefore, it is not possible to optimise a policy response without taking into account health infrastructure, implementation challenges, data system structure, public-private infrastructure and expert opinions where data are not available. It is for this reason that the premise of a central model to optimise a country’s response fails.

Reply: The reviewer raises an important point that highlights the utility of our approach. This work is actually directly motivated by the variable implementation of policies between countries. Policy efficacy is inequitably distributed across countries due to the influence of the issues raised by the reviewer such as public-private health infrastructure and international pressure etc etc. By taking the identity of a country into consideration, many of the complex multi-tiered influencing factors are taken into consideration by the model, without needing to specifically capture or encode them. It is likely more accurate to capture the subtleties of these complex influencing factors in this way rather than by adding a poorly collected estimate.

The attainment of optimisation is dependent on the metrics used to measure the trade-off. We use the reported metrics of each country. The reported metrics are routinely used by policymakers. Our work specifically does not comment on the association between the metric used and the actual ground truth of that metric. That question is something that would likely require large-scale primary data collection and experienced policymakers to deduce. Rather, we estimate how this reported metric (irrespective of its association to reality) would change in light of reported policy changes, given the data.

As many policymakers use these metrics to measure *relative* policy effectiveness (usually with an understanding of their relationship to reality). Our work aligns with the information that policymakers routinely use. Thus it is an analytical scaffold to better understand hidden

patterns in the available, reported data and estimate how this data reacts to changes in policies.

Policy decision support systems like this one are strictly not designed to replace expert opinion. Rather they are designed to assist experts better understand the data on which they base their decisions.

> Taken together, it is clear that policy decision support systems like this one are strictly not designed to replace expert opinion but rather to assist experts better understand the data on which they base their decisions.

We have now made this clear in the manuscript and thank the reviewer for the opportunity to do so.

> ## Discussion
>
> This work explores how the public health policies implemented during the COVID-19 pandemic were related to country-specific patterns in the reported metrics they were presumed to impact. It aims to provide insight into the costs and benefits of public health policies and create an analytical framework for guiding a country-specific optimised policy response. While it was trained on COVID-19 data, the framework

Comment 3:

3. Validating in retrospect
   The finding of the analysis is the the model outperforms policymakers. I ask the authors, how valid such an analysis is in the first place. With the benefit of hindsight of months of data, to outperform actions that were based on little to no local data, lack of preparedness of systems that were gradually developed over time and global pressure for action, seems like a simple task, rather than evidence of superior intelligence of the model. If as the authors say weather is difficult to predict accurately, what chance does the model have when global pressure and scientific trends and politics will play a large role to inform the next set of policies?

Reply: Thank-you for this question. The model is not validated in retrospect. The model is given the same data that would have been available to a policymaker at the time of the policy decision.
I.e. it is not allowed to use “future” data.
This is an important point and is now reiterated in several more parts of the paper

See line 62

> used in each model. Only features collected before (and including) the date of the predicted value are considered in the prediction (thus the model only uses past data to predict into the future). The data considered includes:

Comment 4:

4. Incomplete Data sources
   COVID epidemiology is being characterised only by Re; a function of cases. This is dangerous, as the growth rates and Re do not necessarily take into account testing strategy, local test conditions and test sensitivity and most importantly hospital admissions reported deaths and excess deaths. Training the model on Unemployment data for 29 countries (and that too HICs), and then stating the model to be globally relevant is a very big oversight.

Reply: Here, the reviewer reiterates the issue raised in point 1, regarding the relationship of reported metrics to reality.
As mentioned before, our work does NOT aim or claim to predict how the reported data relates to the true values. That question would likely require large-scale primary data collection and experienced policymakers to deduce. Rather, we estimate how this reported metric (irrespective of its association to reality) would change in light of reported policy changes.

As many policymakers routinely use these metrics to measure *relative* policy effectiveness (usually with an understanding of their relationship to reality). Our work allows policymakers to view patterns within the data that they routinely use.

Our work predicts policy effectiveness for R_E (i.e the *benefit* component of the cost-benefit trade-off) in 116 countries. We additionally perform a targeted reinforcement learning analysis on a subset of 29 countries with available data on the “cost” component.

This work is more than “multi-country” (which could be 2 or 3 countries or 300). Covering countries with 91% of the world’s population, for the main results of our paper, we feel that the term “global” is reasonable and appropriate to define the targeted relevance of this work.

An all-or-nothing definition of “global” is unrealistic for almost every study.

Nevertheless, we hope this work can advocate for the potential value of data collection in low-resource settings.

Comment 5:

5. Future application to COVID
By ignoring vaccination and new treatment options, the impact that changes in testing strategy has on Re, and higher seroprevalence brought on by previous infection and vaccination (hybrid immunity), and most importantly by ignoring severe illness and death, this model is even less useful for future COVID public health modelling. Hybrid immunity means that one can expect less severe COVID (barring new variants with extreme immune loss), and with reduced testing, cases become a less relevant measure on which to base policy changes.

Reply: As stated throughout the manuscript, this work focuses on gauging the efficacy of NON-pharmaceutical measures.
Vaccination and variant severity may affect how people respond to policies, this can be learned by the models without specifically encoding it as a feature and should be seen as a drift in efficacy over time.
Sub analyses of how predicted policy efficacy changes over time and its causal links to vaccination and severity are just another example of exciting avenues of research that could be built on our work.

Comment 6:
The model is not trained on sufficient public health data, mostly because it doesn’t exist. The model does not incorporate contextual knowledge to allow it to be relevant for countries, particularly LMIC. While it may be of theoretical value, its usefulness in public health decision making is unfortunately limited. The manuscript may be better suited for publication in a theoretical statistical journal.

Reply: We thank the reviewer for allowing us to make edits to our manuscript that reveal its enormous practical potential value, as recognised by the first reviewer. Indeed predicting country-level pandemic policy effectiveness in data that is routinely used by policymakers is of great practical relevance. We feel that it is now clear that our work answers a deeply practical question with global-scale potential for follow-up studies. It is thus perfectly suited to the scope of PLoS Global Public Health and written specifically for its audience.

The claim that it does not incorporate contextual knowledge, stems from the misunderstanding that we have now clarified (i.e. that country-specific contextual information can be learned without being specifically featurized).
As stated previously, the main results of this work covers countries representing 90% of the global population.
